# Antioxidant Therapy Significantly Attenuates Hepatotoxicity following Low Dose Exposure to Microcystin-LR in a Murine Model of Diet-Induced Non-Alcoholic Fatty Liver Disease

**DOI:** 10.3390/antiox11081625

**Published:** 2022-08-22

**Authors:** Apurva Lad, Jonathan Hunyadi, Jacob Connolly, Joshua D. Breidenbach, Fatimah K. Khalaf, Prabhatchandra Dube, Shungang Zhang, Andrew L. Kleinhenz, David Baliu-Rodriguez, Dragan Isailovic, Terry D. Hinds, Cara Gatto-Weis, Lauren M. Stanoszek, Thomas M. Blomquist, Deepak Malhotra, Steven T. Haller, David J. Kennedy

**Affiliations:** 1 College of Medicine and Life Sciences, University of Toledo, Toledo, OH 43614, USA; 2 Department of Clinical Pharmacy, University of Alkafeel, Najaf 54001, Iraq; 3 Department of Chemistry and Biochemistry, University of Toledo, Toledo, OH 43606, USA; 4 Department of Pharmacology and Nutritional Sciences, University of Kentucky College of Medicine, Lexington, KY 40506, USA

**Keywords:** non-alcoholic fatty liver disease (NAFLD), microcystin-LR (MC-LR), pNaKtide, n-acetylcysteine (NAC)

## Abstract

We have previously shown in a murine model of Non-alcoholic Fatty Liver Disease (NAFLD) that chronic, low-dose exposure to the Harmful Algal Bloom cyanotoxin microcystin-LR (MC-LR), resulted in significant hepatotoxicity including micro-vesicular lipid accumulation, impaired toxin metabolism as well as dysregulation of the key signaling pathways involved in inflammation, immune response and oxidative stress. On this background we hypothesized that augmentation of hepatic drug metabolism pathways with targeted antioxidant therapies would improve MC-LR metabolism and reduce hepatic injury in NAFLD mice exposed to MC-LR. We chose N-acetylcysteine (NAC, 40 mM), a known antioxidant that augments the glutathione detoxification pathway and a novel peptide (pNaKtide, 25 mg/kg) which is targeted to interrupting a specific Src-kinase mediated pro-oxidant amplification mechanism. Histological analysis showed significant increase in hepatic inflammation in NAFLD mice exposed to MC-LR which was attenuated on treatment with both NAC and pNaKtide (both *p* ≤ 0.05). Oxidative stress, as measured by 8-OHDG levels in urine and protein carbonylation in liver sections, was also significantly downregulated upon treatment with both antioxidants after MC-LR exposure. Genetic analysis of key drug transporters including *Abcb1a*, Phase I enzyme-*Cyp3a11* and Phase II metabolic enzymes-*Pkm* (Pyruvate kinase, muscle), *Pklr* (Pyruvate kinase, liver, and red blood cell) and *Gad1* (Glutamic acid decarboxylase) was significantly altered by MC-LR exposure as compared to the non-exposed control group (all *p* ≤ 0.05). These changes were significantly attenuated with both pNaKtide and NAC treatment. These results suggest that MC-LR metabolism and detoxification is significantly impaired in the setting of NAFLD, and that these pathways can potentially be reversed with targeted antioxidant treatment.

## 1. Introduction

Cyanobacteria (photosynthetic bacteria) are a component of phytoplankton in both freshwater and marine ecosystems in addition to select terrestrial systems [1,2,3]. Overgrowth of cyanobacteria, commonly in Harmful Algal Blooms, are considered a health risk due to the release of cyanotoxins. The increase in abundance of severe blooms has been attributed to both eutrophication and to rising temperatures. Specifically, changing climate conditions have been shown to affect nutrient levels and temperature in freshwater systems, altering cyanobacteria intensity and distribution globally [4].

Production of toxins is a common cyanobacterial characteristic, and microcystins (MCs) are the most widespread of these toxins [5]. The most common microcystin is microcystin-LR (MC-LR). It is a cyclic heptapeptide identified by a leucine and an arginine residue at positions 2 and 4 on the cyclo-(D-alanine-1-X2-D-MeAsp3-Y4-Adda-5-d-glutamate-6-Mdha-7) structure [6]. MC-LR typically enters cells through organic anion transporting polypeptides (OATPs) and impairs them by inhibiting activity of protein phosphatase 1 (PP1) and 2A (PP2A), as well as by increasing reactive oxygen species (ROS) [7,8,9]. The resulting induced hyperphosphorylation and oxidative states promote changes to the cytoskeleton, OATP expression, glycogen storage, mitogen-activated protein kinase (MAPK) activity, and mitochondrial structure and function. Cellular disruption, endoplasmic reticulum dysfunction, DNA damage, inflammation and tumor growth can also occur [10,11,12,13,14,15,16,17,18]. A recent review by Arman and Clarke gives a detailed insight into the molecular toxicity, toxicokinetics and the pathophysiology of MC in both rodent models and humans [19]. It known that oral consumption is the major route of exposure to MC. Apart from liver; intestine and the kidney are the other major targets of MC. Other targets include the nervous, cardiac, pulmonary, and reproductive system. Once absorbed, the toxin covalently binds to and inhibits protein phosphatases leading to an array of downstream toxicities including increase in oxidative stress, cytoskeleton disruption and cell death in the form of apoptosis, necrosis and autophagy. Microcystin toxicokinetics typically involves low absorption in the intestine followed by rapid distribution to liver where it is metabolized via the glutathione pathway forming cysteinyl conjugates which are then expelled via urinary or fecal excretion.

The accumulation of hepatic lipids, or simple steatosis, is a benign condition that is a precursor to that can progress to NAFLD, which is one of the most common causes of chronic liver diseases worldwide. NAFLD is a condition typically characterized by hepatic steatosis, at least 5% fat infiltration, and inflammation, is a widespread liver condition in the United States [20,21,22,23]. NAFLD incidence has roughly doubled in the past 20 years, in correlation with increasing rate of obesity and diabetes, and affects 20–30% of the population in western countries [24]. Hepatic impairment linked to NAFLD and steatosis in hepatocytes can potentially result in the activation of pathways that progress to a worsened state such as non-alcoholic steatohepatitis (NASH) and cirrhosis [25]. Due to the rise in both NAFLD incidence and cyanobacterial blooms, we recently studied the effects of oral MC-LR exposure on pre-existing NAFLD using the murine Lepr^db^/J NAFLD model. We found that chronic oral exposure to MC-LR, beneath the No Observed Adverse Effect Level (NOAEL), worsened liver damage. Exacerbation of liver injury was characterized by oxidative stress, excess micro-vesicular hepatic lipid accumulation, upregulation of multiple hepatotoxicity genes, and alterations in the phosphorylation of cell cycle and immune response regulatory pathways [26]. Importantly, these phenotypic changes were associated with significantly impaired hepatic metabolism and excretion of MC-LR compared to MC-LR exposed healthy mice.

On this background we tested the hypothesis that augmentation of hepatic drug metabolism pathways with targeted antioxidant therapies would improve MC-LR metabolism and reduce hepatic injury in NAFLD mice exposed to MC-LR. Targeted antioxidants included augmentation of the glutathione detoxification pathway with N-acetylcysteine (NAC) and interruption of a specific Src kinase-mediated oxidant signaling pathways with a novel peptide (pNaKtide).

## 2. Materials and Methods

### 2.1. Animals

Five-week-old male C57Bl/6J (Jax Stock No. 000664, Black 6) mice were acquired from The Jackson Laboratory (Bar Harbor, ME, USA) and maintained in the Department of Laboratory Animal Research at University of Toledo. All the mice obtained were of specific-pathogen free and housed in plastic cages (five mice per cage) and fed ad libitum on standard rodent diet (Teklad global 16% protein diet, Envigo, Indianapolis, IN, USA) and water for a week to allow them to acclimatize before beginning the study. The animals were kept in well-ventilated room maintained at 23 °C on a 12-h light and dark cycle. All the protocols were approved by the University of Toledo Institutional Animal Care and Use Committee (IACUC protocol number 108663, approval date 9 February 2016) and conducted in accordance with the National Institute of Health (NIH) Guide for the Cure and Use of Laboratory Animals.

### 2.2. Experimental Design

**Figure 1:**
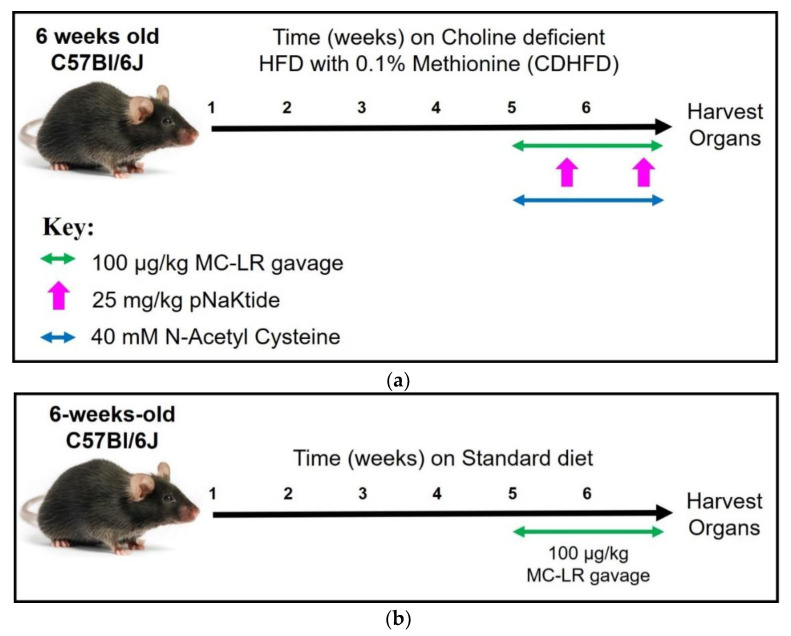
Overview of the study designs to study the effect of antioxidants on low dose exposure to MC-LR with (**a**) choline deficient high fat diet–induced NAFLD and (**b**) healthy C57Bl/6J mice. (**a**) Six-week-old mice were fed on CDHFD for six weeks to induce NAFLD. During weeks 5 and 6, mice were orally gavaged with 100 µg/kg MC-LR every 24 h. Mice in the antioxidant groups were given an I.P. injection of 25 mg/kg pNaKtide once a week (2 doses) or 40 mM NAC in drinking water every day during weeks 5 and 6. (**b**) In a parallel study the mice were fed a standard diet and gavaged with MC-LR during weeks 5 and 6.

At 6-weeks of age, animals were randomly divided into four groups with 5–8 mice per group (Figure 1a). The mice were put on L-Amino Acid Diet with 60 kcal% Fat with 0.1% Methionine and No Added Choline (Research Diets, New Brunswick, NJ, USA), hereafter referred to as the CDHFD (Choline Deficient High Fat Diet), for a period of six weeks. Group 1 was gavaged with water equivalent to that of the MC-LR exposed mice (300 µL); Group 2 was gavaged with MC-LR (Cat. No. 10007188, Cayman Chemicals, Ann Arbor, MI, USA) at 100 µg/kg of body weight; Group 3 was exposed to 100 µg/kg of MC-LR and injected with 25 mg/kg of pNaKtide (synthesized by Ohio Peptide, Powell, OH, USA) [27,28] intraperitoneally (i.p.) once per week during weeks 5 and 6; and Group 4 was exposed to 100 µg/kg of MC-LR via gavage and 40 mM of NAC (Cat. No. 616-91-1, Sigma-Aldrich, St. Louis, MO, USA) in drinking water every day during weeks 5 and 6 [29]. MC-LR or Vehicle was administered via gavage every 24 h (total 15 doses over 2 weeks). As described earlier, these doses approximate the currently accepted NOAEL (40 µg/kg) established over a period of 13 weeks [26,30]. The toxin was freshly prepared by dissolving the lyophilized product in 2 mL of Milli Q water to obtain a working concentration of 0.5 mg/mL. The animals were weighed once a week during the six weeks of the treatment. Blood samples were collected via retro-orbital bleeding (after isoflurane anesthesia) once at the beginning of the study and once at the end of the study for blood chemistry analysis. At the end of the study, animals were placed in metabolic cages for urine collection following the final dose of the treatment for 24 h. The urine samples collected were stored at −80 °C for further use. Mice were placed back into the normal cages and euthanized after another 24 h (48 h after the last exposure). Blood was collected via intracardiac puncture immediately after euthanasia in K3-EDTA microtubes (Sarstedt, Newton, NC, USA). Plasma was separated by centrifuging the samples at 2000 r.p.m./10 min/4 °C. The plasma obtained was separated and stored at −80 °C for further use.

After the animals were humanely euthanized and their blood collected via intracardiac puncture, they were flushed with 10 mL of 1X Phosphate Buffered Saline (PBS) (Fisher Scientific, Pittsburgh, PA, USA). Liver tissues were collected from each animal and weighed. The liver tissue was divided into three parts: one part was frozen in OCT embedding media (Fisher Scientific, Pittsburgh, PA, USA) for Oil Red O (ORO) staining, another was fixed with 4% *w/v* Formalin (Fisher Scientific, Pittsburgh, PA, USA) for histological purposes, and the remainder was snap frozen in liquid nitrogen and stored at −80 °C for further experimental purposes.

In a parallel study (Figure 1b), age matched C57Bl/6J male mice were randomly divided into 2 groups: Vehicle (*n* = 5) and 100 µg/kg MC-LR exposed group (*n* = 5). These mice were fed the standard diet throughout the experiment. The animals were exposed to the appropriate treatment and the tissues obtained from these animals were processed as detailed above.

### 2.3. Nuclear Magnetic Resonance (NMR) Spectroscopy

Nuclear Magnetic Resonance (NMR) Spectroscopy (Bruker, Billerica, MA, USA) was performed on the mice at three different time points–before the start of CDHFD (Day 0 of treatment), end of week 4 (before exposure) and at the end of the study (after treatment) to analyze their total body fat content, lean mass and fluid content. For the purpose of this analysis, the mice were weighed and placed in the imaging tube. Data obtained from this procedure was normalized to the body weight for each individual mouse.

### 2.4. Histology

As mentioned earlier, liver sections were fixed in 4% *w/v* Formalin solution for 24 h, dehydrated in 70% ethanol, embedded in a paraffin block and cut with a microtome to yield 4 μm thick sections. Hematoxylin & Eosin (H&E) staining was then performed on these sections. Hepatic inflammation was analyzed based on the infiltration and aggregation of the immune cells around hepatocytes with large lipid droplets, similar to hepatic crown-like structures (hCLS) [31]. To quantify these foci, images of the H&E-stained liver sections were captured on Olympus VS120 Virtual Slide Microscope (Olympus, Tokyo, Japan) at 20× magnification. The number of hCLS were counted in five fields (Scale = 100 µm) chosen randomly for each section.

### 2.5. Oxidative Stress Analysis

#### 2.5.1. Quantification of 8-Hydroxy 2 Deoxyguanosine (8-OHDG)

Free 8-OHDG levels in the urine of animals were quantified using 8-hydroxy 2 deoxyguanosine ELISA Kit (ab201734 Abcam^®^, Cambridge, UK) by following the manufacturer’s protocol. Fifty 50 µL of undiluted urine was used. Data obtained from this assay was normalized to the 24 h urine volume.

#### 2.5.2. Protein Carbonylation Immunostaining

OCT-embedded liver sections were collected as described earlier (*n* = 3/group, representative) and were stained for protein carbonyl fragments using Protein Carbonyls Immunohistochemical Staining kit (SML-ROIK04-EX, Cosmo Bio, Carlsbad, CA, USA) following the kit protocol. The stained regions were quantified using the Image ProPlus 7.0 (ImageIQ Inc., Cleveland, OH, USA) and Image J (NIH, Bethesda, MD, USA) Softwares. 

### 2.6. RNA Extraction and RealTime—PCR Analysis

Around 10 mg of the snap frozen liver tissue was taken to extract RNA using QIAzol/Chloroform extraction method and ~500 ng of the extracted RNA was taken to synthesize cDNA using QIAGEN’s RT^2^ First Strand Kit (Qiagen, Germantown, MD, USA). Automated liquid handling workflow systems–QIACube HT (for RNA extraction) and QIAgility (for qPCR sample and reagent loading)–were used. qPCR was performed using a Qiagen Rotor-Gene Q thermo-cycler. The cycle threshold values obtained in the process were used to calculate the fold change in the gene expression. We used 18S rRNA (Cat. 4319413E, Thermo Fisher Scientific, San Jose, CA, USA) as a housekeeping gene for normalization. The following Taqman primers obtained from Thermo Fisher Scientific) were used to assess inflammation and hepatotoxicity: *CD40* (Mm00441891_m1), *Itgam* (Mm00434455_m1) and *MMP2* (Mm00439498_m1). The data obtained is from four representative animals from each group. 

### 2.7. Drug Metabolism Array

RNA extraction and cDNA were prepared as described in Section 2.6. Each exposure group shows data from 3 arrays and each array was pooled cDNA from 2 mice/group. Hence, the data is representative of 6 mice from each exposure group. The pooled cDNA was then used to run RT^2^ Profiler^TM^ Mouse Drug Metabolism PCR Array (PAMM-002Z, Qiagen, Germantown, MD, USA) using the Qiagen Rotor Gene Q thermo-cycler. The data was analyzed using Qiagen Geneglobe analysis software (Qiagen, Germantown, MD, USA) and the criteria for fold change was based on any changes that are at least 2-fold above the normalized value for the vehicle-exposed group. The *p* value for significance was considered as *p* < 0.05.

### 2.8. MC-LR and MC-LR Cysteine Determination in Urine

MC-LR and its metabolite, MC-LR Cysteine, in the urine samples were quantified using High-Pressure Liquid Chromatography (Shimadzu Technologies, Addison, IL, USA)–Orbitrap Fusion Mass Spectrometry (Thermo Fisher Scientific, San Jose, CA, USA) as previously described [32,33].

### 2.9. Glutathione-S-Transferase Activity Assay

Total glutathione-S-transferase (GST) activity (both cytosolic and microsomal) was measured in 10 mg of liver tissues using the Glutathione-S-transferase Assay kit (703302, Cayman Chemicals, MI, USA) according to the kit protocol. Data from the activity assay was normalized to protein concentration estimated using the Lowry protein quantification assay. 

### 2.10. Statistical Analysis

Statistical analysis was performed using GraphPad PRISM 7 software (San Diego, CA, USA). Comparison of different experimental groups was performed using Unpaired Student’s *t*-Test and Analysis of Variance (ANOVA). Paired *t*-Test was used to compare the data collected at different time points for the same group. All the data are presented as Mean ± Standard Error of Mean (S.E.M.) and a *p* < 0.05 was considered statistically significant.

## 3. Results

### 3.1. Survival and Weights

None of the animals showed any symptoms of sickness or discomfort throughout the study. No mortality was reported in any of the groups, in contrast with previous studies with Lepr^db^/J mice [26]. In the CDHFD model, mice treated with Vehicle (*n* = 5) did not show any significant change in body weight. On the other hand, mice that were exposed to MC-LR (*n* = 5) showed a significant weight gain throughout the study especially after exposure to MC-LR. Interestingly, mice treated with antioxidants pNaKtide (*n* = 7) and NAC (*n* = 8) showed significant decrease in the body weight after treatment with antioxidants. In the diet control model, both Vehicle and MC-LR exposed mice did not show any significant weight gain after exposure (Supplementary Materials Figure S1a). 

There were also no significant changes in the liver weights, however, in both CDHFD and diet control model, livers of mice exposed to the toxin showed a decreasing trend as compared to the Vehicle (Supplementary Materials Figure S1b).

### 3.2. Assessing Lipid Content—Whole Body and Liver

**Figure 2:**
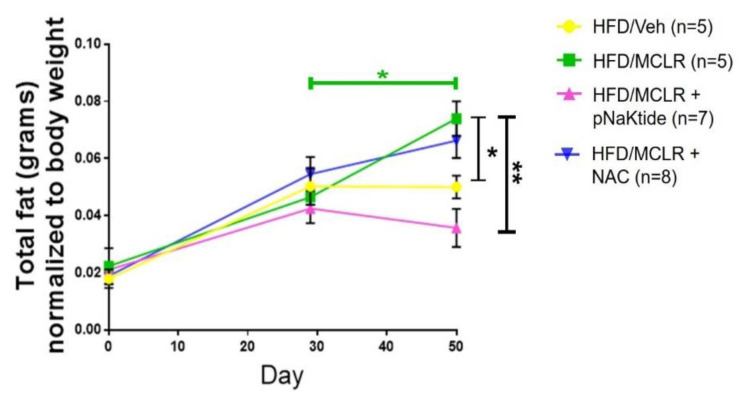
Antioxidant treatment reduces MC-LR induced increases in total body fat: NMR spectroscopy-based analysis revealed reduction in the total body fat content of mice that were treated with the antioxidant pNaKtide after MC-LR exposure in a diet-induced model of NAFLD. * *p* ≤ 0.05, ** *p* ≤ 0.01.

To determine the total lipid content in the body, we used NMR Spectroscopy to quantify the total body fat, lean mass and fluid content of the mice. As shown in Figure 2, CDHFD mice that were exposed to MC-LR showed significant fat accumulation in their body after the exposure as compared to Vehicle mice. Treatment with antioxidant pNaKtide showed decrease in the fat content after exposure to MC-LR. On the other hand, treatment with NAC did not show any significant change in the lipid content after exposure to the toxin. As seen in Figure 2 (green bar of significance), total lipid content significantly rises after exposure to MC-LR without any treatment with antioxidants.

No significant changes were observed in either Vehicle or MC-LR exposed diet control mice (Supplementary Materials Figure S2). 

In an initial study, livers from Vehicle and MC-LR exposed mice were embedded in OCT mounting media to carry out Oil Red O (ORO) staining. Quantification of the micro and macro-vesicular lipid accumulation revealed significant increase in the lipid content in the MC-LR exposed mice livers (Supplementary Materials Figure S3a,b). Representative pictures of ORO-stained liver sections are shown in Supplementary Materials Figure S3c.

### 3.3. Liver Histology

**Figure 3:**
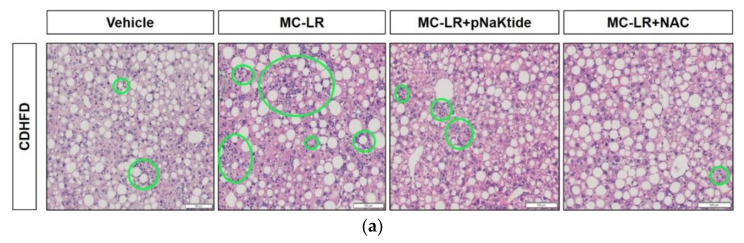

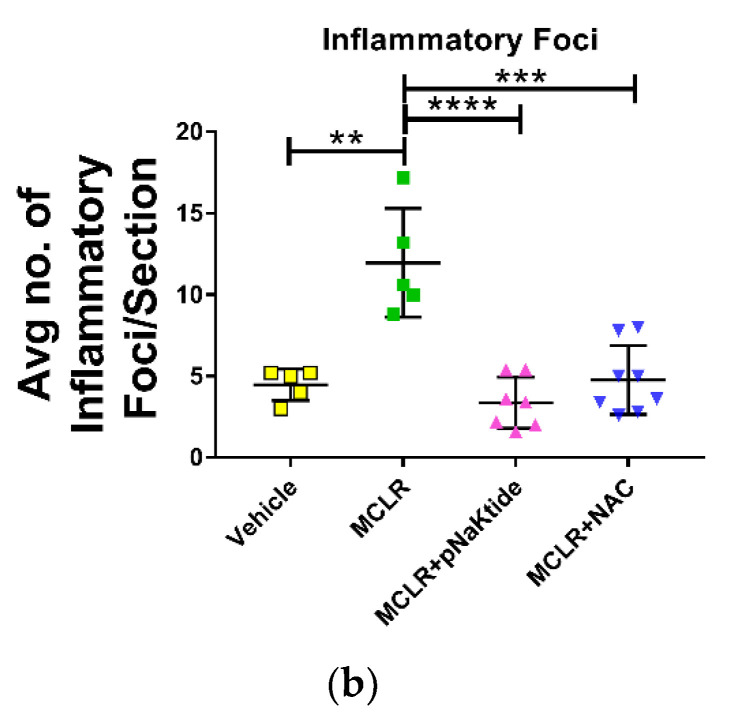
Targeted antioxidant treatment with pNaKtide and NAC significantly reduces MC-LR induced lobular inflammation and apoptotic hepatocytes: (**a**) H&E staining of the liver tissues from diet-induced NAFLD mice treated with antioxidants showed reduced inflammatory foci (circled in green) as compared to those exposed to MC-LR alone (scale bar, 100 μm); (**b**) Quantification of these inflammatory foci showed significant reduction of infiltrating immune cells upon treatment with antioxidants. ** *p* ≤ 0.01, *** *p* ≤ 0.001, **** *p* ≤ 0.0001.

Histopathological analysis was performed on liver sections using H&E staining. As seen in Figure 3, significant immune cell infiltration and aggregation was observed in the livers of CDHFD mice that were exposed to MC-LR as compared to their vehicle treated counterparts (Figure 3a). In the same model, mice treated with antioxidants pNaKtide, and NAC showed significant decrease in infiltrating immune cells (Figure 3a). The inflammatory foci (circled in green) were quantified in five fields selected randomly for each liver section. Quantification showed the significant increases in inflammation in MC-LR exposed livers as compared to non-exposed Vehicle livers. These were significantly reduced on treatment with antioxidants (Figure 3b). No inflammation was observed in the livers of either Vehicle of MC-LR exposed Diet Control mice (data not shown).

### 3.4. Gene Expression in Liver

**Figure 4:**
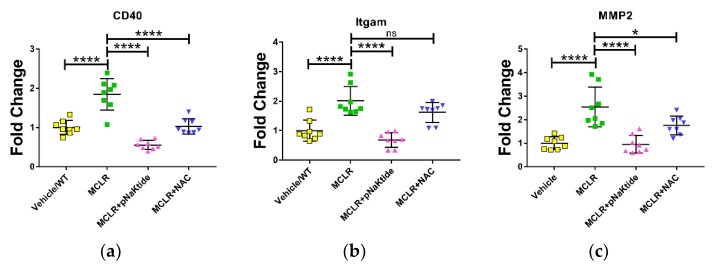
Targeted antioxidant therapy with pNaKtide and NAC significantly downregulates markers of hepatotoxicity. Quantitative PCR (qPCR) analysis of markers of hepatotoxicity such as (**a**,**b**) *CD40* and *Itgam*, also known as *MAC1*, that are known markers of inflammation and (**c**) *MMP2*, a known marker of fibrosis, revealed significant upregulation on exposure to MC-LR which was significantly downregulated with antioxidant treatment (*n* = 4). ns—not significant, * *p* ≤ 0.05, **** *p* ≤ 0.0001.

Studies have shown that exposure to MC-LR is associated with increase in inflammation and fibrosis. We tested a few markers associated with hepatotoxicity (inflammation and fibrosis) with four representative liver samples from each group. Expression of *CD40*, a marker associated with apoptosis and liver damage was significantly upregulated on exposure to MC-LR but significantly downregulated on treatment with antioxidants (Figure 4a). Next, we tested the expression of Integrin Alpha M (*Itgam*), also known as *MAC1*, a well-known marker of inflammation. *Itgam* expression was significantly upregulated on MC-LR and downregulated on treatment with antioxidants (Figure 4b). We also analyzed the expression of Matrix Metalloproteinase 2 (*MMP2*), a known marker of fibrosis, and observed the same trend (Figure 4c). These results indicate that MCLR induced hepatotoxicity can be reduced by antioxidant therapy.

### 3.5. Assessment of Oxidative Stress Markers

Since MC-LR is known to increase oxidative stress, we wanted to determine this phenomenon quantitatively, and we therefore assessed 8-OHDG levels in urine and protein carbonyl fragments in the liver, both of which are well-established markers of oxidative stress. 

**Figure 5:**
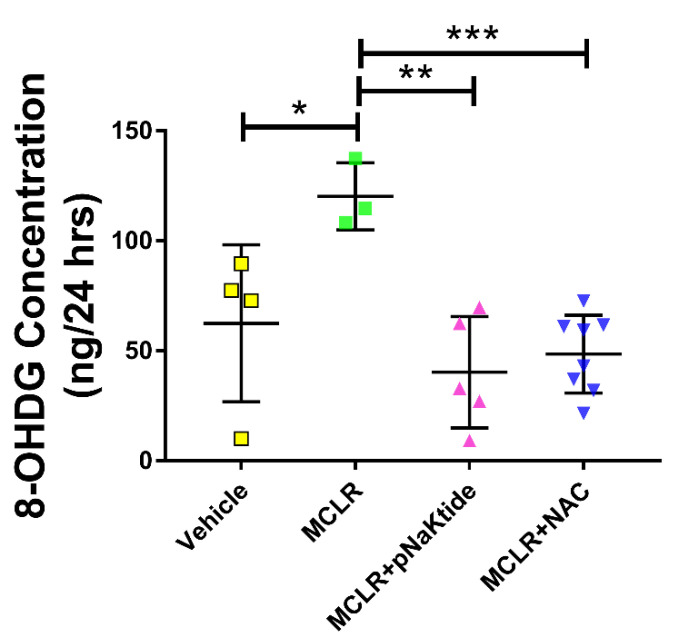
Oxidative stress is significantly reduced after antioxidant treatment. 8-OHDG levels in urine were elevated on exposure to MC-LR and reduced after treatment with antioxidants. * *p* ≤ 0.05, ** *p* ≤ 0.01, *** *p* ≤ 0.001.

We quantified the free 8-OHdG, an oxidative derivative of guanosine, excreted in the urine that was collected over a period of 24 h at the end of the treatment. We observed significant increase in the 8-OHdG levels in mice that were exposed to the toxin alone as compared to Vehicle. Treatment with targeted antioxidant therapy after exposure to the toxin showed significant decrease in the 8-OHdG levels as compared to MC-LR exposed mice (Figure 5).

Immunostaining the protein carbonyl fragments, a typical ROS-induced protein modification, in the livers showed a trend that depicted increase in oxidative stress on exposure to MC-LR but decreased on treatment with targeted antioxidant therapy (Supplementary Materials Figure S4). These results indicate that MC-LR induced oxidative stress could potentially be overcome with antioxidant therapy. 

### 3.6. GST Activity in the Liver

**Figure 6:**
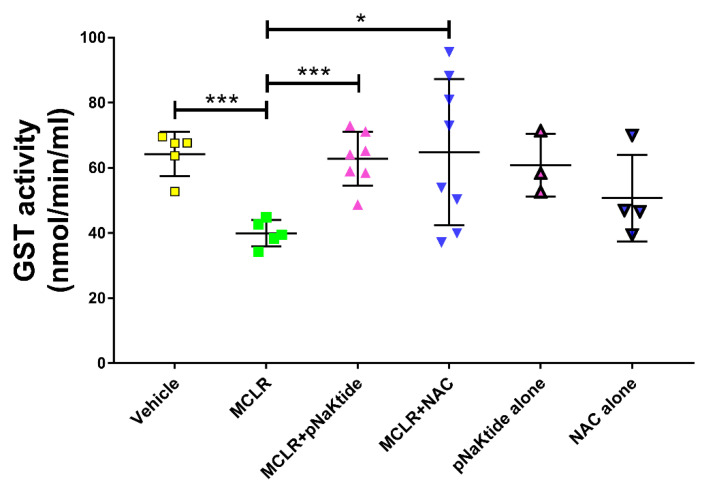
Exposure to MC-LR significantly reduces Glutathione-S-transferase (GST) activity. GST activity was significantly reduced in CDHFD mice exposed to MC-LR but was restored on treatment with targeted antioxidant therapy using pNaKtide or NAC. Treatment of CDHFD mice with antioxidant alone did not alter the enzyme activity. * *p* ≤ 0.05, *** *p* ≤ 0.001.

Glutathione-S-transferases (GSTs) are a family of metabolic enzymes that catalyze the conjugation of reduced glutathione to xenobiotics for detoxification via the glutathione pathway. Assessment of the GST activity in frozen liver samples revealed significantly reduced GST levels in mice that were exposed to the toxin. However, treatment with antioxidants brought the levels up to those of Vehicle treated mice. To verify if the antioxidants alone in the absence of MC-LR exposure had any effect on GST activity, we assessed the same in the livers of mice that were fed the CDHFD and received the same doses of antioxidants as mentioned earlier. It was observed that treatment with antioxidants alone did not alter the levels significantly (Figure 6). DC mice exposed to the toxin did not show any significant changes in the GST activity as compared to the non-exposed Vehicle control mice (Supplementary Materials Figure S5). 

### 3.7. Mass Spectrometric Analysis for MC-LR and MC-LR-Cysteine in Plasma and Urine

In order to determine the circulatory and excretory levels of MC-LR and its metabolite MC-LR-Cysteine after exposure, we collected plasma and 24 h urine samples towards the end of the study. High-Performance Liquid chromatography–orbitrap mass spectrometry (HPLC-orbitrap-MS) was used to detect and quantify the MC-LR and MC-LR-Cysteine (a metabolite of MC-LR degradation through the glutathione detoxification pathway). 

**Figure 7:**
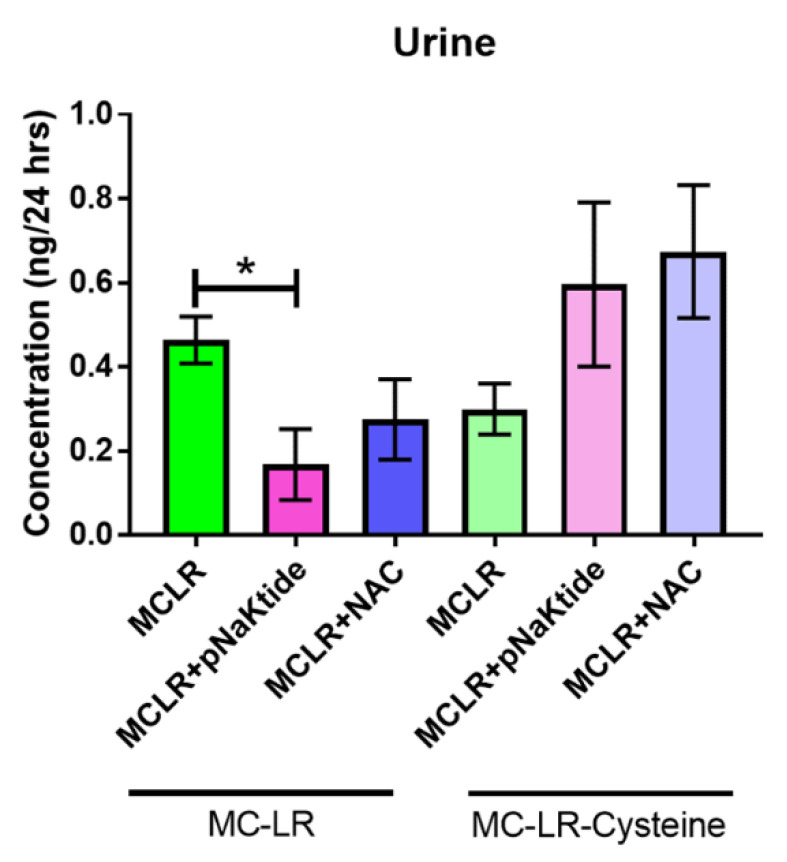
Presence of NAFLD affects MC-LR metabolism. Mass spectrometric analysis of the urine samples revealed that antioxidant therapy with pNaKtide and NAC in the setting of diet-induced NAFLD improved hepatic metabolism of MC-LR to favor detoxified MC-LR-Cysteine metabolite. * *p* ≤ 0.05.

We observed that MC-LR significantly decreased in the urine of mice that were treated with pNaKtide, and those treated with NAC also showed a reduction in MC-LR levels. On the other hand, MC-LR-Cysteine levels were much higher in the antioxidant treated mice as compared to the MC-LR exposed mice (Figure 7). Interestingly, circulating levels of both MC-LR and MC-LR-Cysteine were undetectable in the plasma of all the mice. This data indicates the involvement of the glutathione pathway in the degradation and detoxification of the toxin.

### 3.8. Genetic Analysis of Drug Metabolic Enzymes

**Figure 8:**
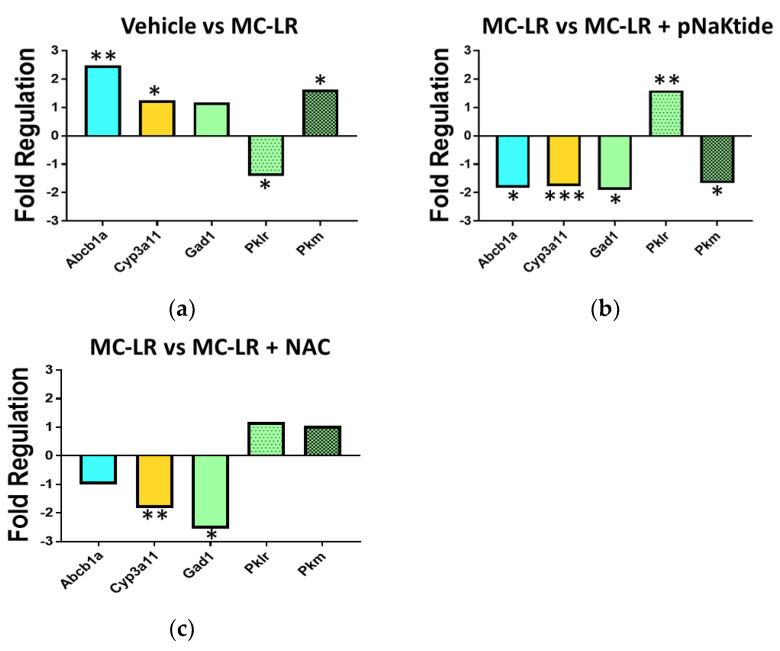
Diet-induced NAFLD mice exposed to low levels of MC-LR affects the expression of Phase I and II enzymes. Quantitative PCR (qPCR) analysis of key drug transporters as well as Phase I and II enzymes revealed significant fold regulation on exposure to MC-LR (**a**) which was reversed by antioxidant treatment using pNaKtide (**b**) and NAC (**c**). * *p* ≤ 0.05, ** *p* ≤ 0.01, *** *p* ≤ 0.001.

Since the GST activity assay and mass spectrometric results showed the involvement of the glutathione pathway, a Phase II metabolic enzyme, we next wanted to assess how the HFD, and antioxidant therapy affected drug metabolism in the presence of NAFLD. We analyzed the genetic expression of some of the major drug transporters and Phase I and Phase II enzymes. Feeding the mice on CDHFD alone significantly downregulated the Phase I metabolizing enzymes (*Cyp17a1*, *Cyp1a2*, *Cyp4b1*, *Cyp2c29*, *Cyp2e1*) and changed the regulation of various classes of Phase II enzymes including glutathione peroxidases (↑upregulated—*Gsta1*, *Gstm3*, *Gpx2*, *Gpx3*, *Mpo*; downregulated—*Gsta3*, *Mgst1*, *Gstz1*); kinases (upregulated—*Hk2*, *Pkm*, downregulated—*Pklr*); oxidoreductases (upregulated—*Aoc1*, downregulated—*Cyb5r3*, *Srd5a1*, *Blvra*, *Nos3*) and glutathione-S-transferases (upregulated—*Gstm3*, downregulated—*Gstt1*). These results are tabulated in Supplementary Materials Tables S1 and S2. Next, we analyzed if treatment with antioxidants after exposure to MC-LR in the setting of NAFLD changes the key enzymes involved in metabolism. Exposure of NAFLD mice to MC-LR significantly upregulated the expression of drug transporter *Abcb1a* by 248% as compared to Vehicle. *Cyp3a11*, a Phase I enzyme belonging to the Cytochrome P450 family, was also significantly upregulated by 125% in the MC-LR exposed group vs. Vehicle whereas Phase II enzymes, *Pkm* (Pyruvate kinase, muscle) was upregulated by 163% and *Pklr* (Pyruvate kinase, liver, and red blood cell) and *Gad1* (Glutamic acid decarboxylase) were significantly downregulated by 142% and 117%, respectively (Figure 8a). Antioxidant therapy with both pNaKtide and NAC significantly reversed these changes vs. MC-LR and restored microcystin detoxification as seen in Figure 8b,c and Supplementary Materials Tables S1 and S2. DC mice exposed to the toxin did not show any major changes in the gene expressions (Supplementary Materials Tables S1 and S2). 

## 4. Discussion

In our previous studies we described that prolonged low dose exposure to MC-LR in the Lepr^db^/J genetic model of NAFLD resulted in increased micro vesicular lipid accumulation in the liver, increased excretion of the toxin as compared to the circulating levels and increased expression of genes associated with hepatotoxicity and oxidative stress [26]. However, genetic models are very pathway specific and are not representative of the complex state of obesity in humans [34]. In this study we used a mouse model in which NAFLD was induced by feeding a diet rich in fat and deficient in choline for 6 weeks. This is a well-established model for better understanding human liver steatosis and to develop potential therapeutics [35]. We observed that there was a significant increase in the body weights of the mice during the first 4 weeks of the study. During weeks 5 and 6 of the study, the mice were either exposed to Vehicle or low dose of MC-LR or were treated with antioxidants. It was observed that CDHFD mice exposed to MC-LR alone continued to gain significant body weight. In contrast, CDHFD mice that were given Vehicle (water) showed a slight but insignificant increase in body weight. In the same study, mice that were treated with the antioxidants pNaKtide or NAC showed significant reduction in body weight. In the parallel diet-control study, mice exposed to vehicle or MC-LR showed an initial increase in weight gain, but no significant change in body weight on exposure to MC-LR. This indicates that our NAFLD model may have an altered metabolism of MC-LR.

There are a wide variety of research models used to study steatosis. These models can be divided into two main categories–genetically induced or acquired via dietary or pharmacological manipulation [36]. Our previously used *db/db* model is an example of the genetically modified leptin resistant model of NAFLD. These mice have normal to higher levels of the leptin but are resistant to its effect because of the insertion of a sequence at the 3” end of the mRNA transcript of the leptin receptor. This insertion sequence contains a stop codon that pre-maturely terminates the leptin receptor leading to loss of function and subsequent leptin resistance [37]. The leptin hormone is involved in various physiological processes and directly affects the inflammatory response. It should be noted that physiological conditions/phenotype created in these mice is not prevalent to humans and is not an accurate correlation to the development of NAFLD/NASH conditions. The Methionine-choline deficient diet (MCD) is a classic diet-induced model of NASH. This diet is high in sucrose (40%) but lacks both methionine and choline which is essential for hepatic mitochondrial β-oxidation and very low-density lipoprotein (VLDL) synthesis. This diet is easy to obtain and use and animals on this diet rapidly develop steatosis [38]. A study carried out by Kirsch et al. to study the effects of MCD diet on different strains of mice and rats showed that male C57Bl/6 mice developed the most inflammation and other hepatic injury [39]. Similar findings were reported in A/J and C57Bl/6 mice strains with A/J showing significantly higher alanine aminotransferase levels and rapid weight loss as compared to the C57Bl/6 mice [40]. However, the main drawback of this dietary model is that their metabolic profile is opposite to that of humans along with an increased risk of mortality [38]. On the other hand, the choline deficient, L-amino acid (CDAA) defined dietary model overcomes the shortcomings of the MCD diet by causing steatosis, fibrosis and liver cancer without the extreme weight loss and mimics the human NASH in both rats and mice. It was noted that although the CDAA diet showed rapid and significant increase in fibrosis and markers of liver injury in rats, these effects were minimal or negligible in mice. Other common dietary models includes high fat, high fructose and high cholesterol diets, however, these are long term diets and it takes at least six months to develop moderate macro-vesicular steatosis and lobular inflammation in C57Bl/6 mice [41]. C57Bl/6 is the most commonly used and well established strain of mice to study metabolic disorders such as NAFLD and NASH [41]. Therefore, Matsumoto et al., showed that formulating a high fat diet (with 60 kcal% fat) deficient in choline and supplementing it with 0.1% methionine demonstrates progression towards steatosis and lipid accumulation over 6 weeks in C57Bl/6J mice, closely modeling human pathophysiology [35], and thus was chosen for the current study.

In the current study we used both NAC and pNaKtide in order to augment MC-LR metabolism in the setting of NAFLD. In vivo NAC can have a direct effect against some ROS, as well as an indirect effect by acting as a precursor to cysteine. In turn, cysteine is a reactant in the rate-limiting step of the production of glutathione, a common direct antioxidant. Additionally, NAC can dismantle disulfide bridges, building thiol pools which balance the redox state [42]. NAC treatment has widely been shown to reduce hepatoxicity by reducing oxidative stress and numerous studies demonstrate its effectiveness in the context of NAFLD [43,44,45,46]. The targeted peptide inhibitor pNaKtide has been successfully used in experimental NAFLD to interrupt amplification of oxidant stress via the Na/K-ATPase/Src kinase signaling cascade pathway [47]. Thus, taken together with the NAC data, our results with pNaKtide indicate that chronic low dose exposure to MC-LR may exhibit toxicity via a Na/K-ATPase/Src kinase signaling pathway and suggests a potential therapeutic target to be explored in future studies. 

MC-LR is a well-known hepatotoxin produced by photoautotrophic cyanobacteria and has been shown to increase lipid accumulation [26]. In support of this we first conducted a pilot study in which livers of mice fed on CDHFD and gavaged with either Vehicle or MCLR were stained with ORO to quantify lipid accumulation. Interestingly, we observed significantly excess accumulation of lipids in the form of micro and macro-vesicular lipid droplets in the livers of mice exposed to the toxin as compared to Vehicle treated ones. In this study, we conducted NMR Spectroscopy on mice at different time points to assess fat accumulation in the body. The initial analysis was conducted before the start of the study to obtain baseline values. The second analysis was conducted after 4 weeks on high fat diet but before exposure to MC-LR or treatment with antioxidants and the third analysis was performed at the end of the study. Mice fed on CDHFD showed significant increase in fat content during the first 4 weeks of the study, however, only those exposed to MC-LR showed significant increase in fat content as compared to the vehicle group. On the other hand, mice exposed to MC-LR in the diet control group did not show significant increase in fat content as compared to the Vehicle. This indicates that MC-LR contributes to increased fat accumulation in mice with NAFLD. These findings are in agreement with our previously reported studies of dose-dependent micro and macro-vesicular lipid accumulation in the livers of genetically modified NAFLD mice models that were exposed to low doses of the toxin [26]. Treatment with pNaKtide was effective in bringing down the fat content significantly. Sedan et al. have also shown the accumulation of fat in the livers of normal mice that were exposed to low doses (50 µg MC-LR/kg or 100 µg MC-LR/kg body weight) of the toxin given every 48 h. for a month [48]. In another study by He et al., the researchers gavaged normal Balb/c mice with 0, 40 or 200 µg MC-LR/kg of body weight every 2 days for 90 days and found that the toxin significantly downregulated the expression of enzymes associated with catalyzing fatty acid β-oxidation. Reduction in the activity of these enzymes in turn enhanced the excess accumulation of lipid in the liver resulting in lipotoxicity and micro-vesicular steatosis [49]. These findings confirm that MC-LR promotes fat accumulation in the liver as well as the fat pads in the body.

Macrophages are known to play a crucial role in chronic inflammatory conditions such as NAFLD and NASH. It has been reported that the unique histological feature termed as Hepatic crown-like structures (hCLS) is indicative of hepatic steatosis. In this phenomenon, CD11c+ macrophages aggregate to surround hepatocytes containing large lipid droplets such as in the adipose tissue. Increased hCLS is known to be an indicator of increased inflammation and fibrosis in steatosis [31]. In this study, we observed a similar immune cell infiltration and aggregation in which CDHFD mice that were exposed to MC-LR showed significantly higher number of inflammatory foci as compared to Vehicle whereas those treated with the antioxidants showed significant reduction in inflammatory foci. Studies have shown the infiltration of immune cells such as macrophages, neutrophils and activation of the Kupffer cells in the liver tissues of mice and rats that were exposed to MC-LR [50,51]. He et al. has described that apart from inhibition of β-oxidation pathway, immune cell infiltration, inflammation, and fibrosis due to prolonged exposure to MC-LR are prominent processes observed in mouse hepatocytes [49]. In our previous study we showed the different pathways affected in livers of mice that were exposed to different doses of the toxin using the phosphoproteomic TiO_2_ labeling approach [26]. Based on this knowledge we tested the genetic expression of a few markers of hepatotoxicity. CD40 is heavily involved in inflammation and expressed by a variety of immune cells such as B cells, dendritic cells, and macrophages as well as non-immune cells such as endothelial, epithelial, platelets, and mesenchymal cells [52,53]. CD40 expression is associated with antigen presentation, B cell proliferation, and T cell activation [54]. We observed that CD40 expression was upregulated in the livers of mice exposed to the toxin indicating increase in inflammation which was significantly reduced on treatment with antioxidants. Another marker associated with inflammation, Itgam or Integrin αM, also showed a significant upregulation on exposure to MC-LR and significant downregulation on treatment with targeted antioxidant therapy. Itgam is an integrin expressed on monocytes and macrophages and is involved in cell adhesion during immune reactions [55]. In another study conducted by Sevastianova et al., the authors have noted that increased expression of Itgam was associated with macrophage infiltration, adipose tissue inflammation, and increased liver fat content [56]. This is in agreement with our histological as well as gene expression findings. Fibrosis is another key feature of liver damage and is mostly associated with NAFLD progression. Acute exposure to MC-LR has been shown to induce hepatic tissue remodeling and fibrosis in humans [57]. In our study, we observed significant upregulation of the matrix metalloproteinase 2 (MMP2), a marker of tissue remodeling, on exposure to MC-LR and was significantly downregulated on treatment with targeted antioxidant therapy including pNaKtide and NAC although we did not observe any histological evidence of fibrosis in the livers. These findings confirm that exposure to MC-LR increases the risk of inflammation and precursors of fibrosis in the livers of mice with NAFLD and that treatment with antioxidants such as pNaKtide and NAC could potentially be used to reverse the adverse-effects induced by the toxin.

We have previously identified pathways that are potentially affected by exposure to MC-LR, and oxidative stress was one of the major outcomes of MC-LR exposure [26]. In this study we wanted to quantify the outcome of oxidative stress and determine whether the antioxidants–pNaKtide and NAC help in reducing this oxidative stress. We first performed 8-OHdG assay with urine samples which is an established marker of oxidative stress. The oxidative derivative of guanosine, 8-hydroxy-2-deoxy Guanosine (8-OHdG), is produced by the oxidative damage of DNA by reactive oxygen and/or nitrogen species. This process occurs in response to both normal metabolic processes and a variety of environmental factors that may increase reactive oxygen and nitrogen species. Increased levels of 8-OHdG are associated with the aging process as well as with a number of pathological conditions including cancer, atherosclerosis, diabetes, and hypertension [58,59]. In our study we observed that the levels of 8-OHdG were significantly higher in mice that were exposed to the toxin as compared to Vehicle. These levels were significantly lowered in mice that were treated with the antioxidants. These suggests that antioxidants might be useful for treating or preventing NAFLD and the deleterious outcome worsened by microcystin, which antioxidants have been shown potentially useful for treating metabolic-induced fatty liver and adiposity [60,61,62,63]. Furthermore, we also examined the phenomenon of protein carbonylation in the liver tissues. Protein carbonylation is defined as non-specific oxidization of the protein side chains of cystine, histidine and lysine residues into aminoacyl carbonyls. This is a typical ROS induced phenomenon and is considered as the major hallmark of oxidative stress because of the harmful irreversible transformations which are chemically very stable allowing for their detection and quantification [64,65]. In our study, we performed immunostaining of the liver tissues and observed elevated levels of protein carbonylation in the mice that were exposed to the toxin as compared to Vehicle and these levels were lowered upon treatment with targeted antioxidant therapy. Previously we have reported significant increases in protein carbonyl levels in both liver and intestines of tadpoles exposed to 1 µg/L of MC-LR for just 7 days [66]. In another interesting study conducted by Hwang et al., the authors have reported that repeated MCLR exposure potentiates Kainic Acid-induced excitotoxicity in the hippocampus by enhancing oxidative stress and neuroinflammation through the modulation of p-CaMKII, p-PKC and p-ERK [67].

Microcystins are secondary metabolites that can be degraded via biotic or abiotic processes. Glutathione is a common peptide involved in the degradation and detoxification of xenobiotic products. In a review by Schmidt et al., the authors have elaborated on the glutathione metabolic pathway on microcystin degradation. [68]. Our study shows significantly lower levels of GST activity in the MC-LR exposed livers as compared to the Vehicle and these levels were restored when treated with antioxidants. Treatment of animals fed on CDHFD and treated with antioxidants alone did not alter the GST levels indicating the reduction in GST activity was a response to MC-LR, which is a well-known pro-oxidant molecule [19,26,57,69]. In a pharmacokinetics/pharmacodynamics (PK/PD) study carried out by Gehringer et al., it was reported that mice exposed to 75% LD_50_ dose of MC-LR did not show any significant changes in the soluble GST levels in the liver over the 32 h. observation period although there was an increase in levels at 8 and 16 h. post-exposure but not statistically significant [70]. In an interesting study conducted by Li et al., the authors investigated the role of glutathione and its related enzymes in healthy Sprague-Dawley rats with MC-LR induced hepatotoxicity [71]. The rats were given an i.p. injection of 0.25 LD_50_ or 0.5 LD_50_ of MC-LR and Buthionine-(*S*,*R*)-sulfoximine (BSO) was used as the glutathione inhibitor. Analysis of the genetic expression of GST revealed significant upregulation (1.8-fold) in its transcription on exposure to 0.25 LD_50_ but no change when exposed to 0.5 LD_50_ of MC-LR. In agreement with these findings, we did not observe any significant changes in the GST activity in the livers of Diet Control mice exposed to either Vehicle or MC-LR. NAFLD, however, is the metabolic manifestation of a metabolic syndrome. Oxidative stress in NAFLD conditions is well known to cause direct damage to lipids, proteins, and DNA which initiates inflammatory and fibrogenesis pathways further leading to progression of NAFLD to NASH [72,73]. It has been noted that oxidative stress in the setting of NAFLD/NASH can lead to overproduction of reactive oxygen species that can deplete antioxidants such as glutathione and inhibit antioxidant enzymes such as superoxide dismutase, GST, etc. In our study, we observed increase in the GST activity of mice that were fed with CDHFD as compared to the healthy mice fed a normal diet. This could be a response to overcome the steady increase in steatosis. In a study performed by Jayaraj et al. [74], the mice were intraperitoneally injected with either 0.5 LD50 (38.31 µg/kg) or 1 LD50 (76.62 µg/kg) of MC-LR and the biochemical variables were determined at various time points over a period of 7 days. It was observed that there was a significant increase in the liver-body weight index, hepatic lipid peroxidation as well as depletion of GSH levels in mice that received 1 LD50 MC-LR as compared to the Control. A significant decrease in the activity of antioxidant enzymes such as glutathione peroxidase (GPX), superoxide dismutase (SOD), catalase (CAT), glutathione reductase (GR) and glutathione-S-transferase (GST) was also reported in the 1LD50 group. These observations indicate that additional stress or a second hit induced by MC-LR might inhibit the GST activity causing a significant decrease in our model. Treatment with pNaKtide or NAC, both of which are known to augment the glutathione pathway, restore the depleted hepatic antioxidant enzyme activity. A similar study performed in fish that were pre-treated with NAC followed by exposure to Dichlorvos (2,2-dichlorovinyl dimethyl phosphate, DDVP), an organophosphorus (OP) insecticide used in fish farming, showed reduction in the GST activity on exposure to the insecticide which was restored on treatment with NAC [75]. We also demonstrated that the levels of the toxin and its metabolite MC-LR Cysteine could be detected in the urine using the mass spectrometric approach and the results are in line with GST activity such that MC-LR levels were seen to be significantly decreased on treatment with antioxidants whereas there was an increasing trend in the levels of MC-LR Cysteine indicating that NAFLD along with MC-LR causes reduction in glutathione and its related enzymes that affects the metabolism of the toxin which is restored by treatment with antioxidants.

The liver is the most important organ involved in the metabolism of toxins, drugs, and other chemicals that are usually activated or mostly inactivated in the liver [76]. Complex metabolic conditions such as NAFLD can have varied influences on drug transporters and the Phase I and II enzymes due to associated pathological and physiological changes [61,77,78]. For example, inflammatory conditions have been shown to down regulate expression and activity of drug transporters as well as the metabolizing enzymes [79,80]. Oxidative stress caused by NAFLD activates the *Nrf2* transcription factor which in turn activates the Phase I and II enzymes associated with oxidative stress response [61,81,82,83,84,85]. Detoxifying pathways in the liver are important for the metabolism of xenobiotics [77,81,82], which includes uptake or efflux drug transporters to bring the compounds into hepatocytes for clearance. The uptake transporters mainly belong to the solute carrier superfamily and facilitate the compound to be taken up by the cell. Uptake transporters include OATPs (organic anion transporting polypeptides), OCTs (organic cation transporter), and OATs (organic anion transporter) whereas the efflux transporters belong to the ABC (ATP-binding cassette) superfamily. Studies carried out by Canet et al. in rodents with either diet-induced or genetically modified for NASH showed induction of efflux transporters and repression of uptake transporters in the liver mRNA and protein expression [86,87]. In our study, diet induced NAFLD mice showed significant upregulation in efflux transporters as compared to healthy mice. A second hit with exposure to MC-LR also led to significant upregulation of the efflux transporter *Abcb1a* which was significantly downregulated when treated with antioxidants-pNaKtide and NAC. On a second note, MC-LR did not alter the expression of any drug transporters in healthy mice. Once inside the cell, the toxin undergoes metabolism by Phase I and II enzymes. Phase I enzymes belong to the cytochrome P450 (*CYP*) family and mainly carry out oxidative processes [79]. There are 18 known families of *CYP* enzymes but only a few are known to be involved in the metabolism of xenobiotics [88]. Numerous studies have been performed to interpret the role played by *CYP* enzymes in metabolism of xenobiotics in NAFLD conditions and the results reported are conflicting. This could be because of the variety of models used to study the metabolic condition. In our study, we found that mice with NAFLD showed downregulation of the various *CYP 1*, *2* and *4* enzymes as compared to the healthy Vehicle mice. Healthy mice exposed to the toxin did not show any significant alterations in the Phase I enzymes as compared to the healthy Vehicle treated mice, however, NAFLD mice exposed to the toxin showed significant upregulation of *Cyp3a11* which was downregulated on treatment with targeted antioxidant therapy. Although many studies report conflicting results regarding the expression and activity *CYP3A* under NAFLD settings. Zhang et al. conducted a study to determine the effect of MC-LR on the expression of cytochrome P450 isozymes (*CYP1A1*, *CYP2E1* and *CYP3A11*) where mice were injected intraperitoneally with 2, 4 and 8 µg MC-LR/kg body weight for seven days [89]. It was noted that the transcription of the *CYP* genes was initially suppressed but significantly upregulated after seven days of exposure. Phase II enzymes are a group of enzymes that are involved in the conjugation and detoxification (collectively referred to as biotransformation) of the xenobiotic substrates into inactive, non-toxic form that is easily excreted out of the body. Phase II enzyme families include decarboxylases, glutathione peroxidases, glutathione-S-transferases, kinases, etc. among others. As stated earlier, GSTs play a significant role in reducing oxidative stress and the presence of NAFLD has been reported to deplete the antioxidant leading the progression to NASH. GSTs are present as different isoforms–alpha, mu and pi that help in conjugating the reduced GSH to the drug/toxin [76,78,90,91]. Our study showed that presence of NAFLD significantly altered the expression of various Phase II enzymes as compared to the healthy livers. MC-LR exposure in healthy animals altered the expression of few glutathione peroxidases but no other enzymes were altered. Exposure to MC-LR in NAFLD conditions significantly altered the expression of Pyruvate kinases (*Pklm*, *Pklr*) which were reversed on treatment with targeted antioxidant therapy.

## 5. Conclusions

In conclusion, the presence of pre-existing conditions such as NAFLD affects the ability of the liver to metabolize xenobiotic compounds such as MC-LR thus, making patients with NAFLD susceptible to excess inflammation and oxidative stress even at doses lower than the established NOAEL. In our study, we have demonstrated that treatment of diet-induced NAFLD mice exposed to low doses of MC-LR for a short period of time with targeted antioxidant therapy can help lower inflammation and immune cell infiltration into the liver as well as reduce the effects of oxidative stress observed in the form of reduced 8-OHdG in urine. We also demonstrated that treatment with antioxidants helped in restoring the GST levels that were depleted on exposure to the toxin. Mass spectrometric analysis revealed improvement in the detoxified metabolite levels of the toxin as quantified in the urine obtained from mice that received the antioxidant therapy. Additionally, we also studied the effect of NAFLD and MC-LR exposure on drug transporters and drug metabolism enzymes and noted that presence of NAFLD significantly alters the ability of the liver to detoxify and metabolize xenobiotic compounds such as MC-LR and treatment with the antioxidants pNaKtide and NAC reversed these changes. All these findings indicate that targeted antioxidant treatment could be utilized as a potential therapeutic approach to overcome the adverse-effects of MC-LR exposure within the setting of pre-existing NAFLD.

## Data Availability

The datasets generated and/or analyzed during the current study are contained within the article and supplementary data and available from the corresponding author on reasonable request.

## Supplementary Materials

The following supporting information can be downloaded at: https://www.mdpi.com/article/10.3390/antiox11081625/s1, Figure S1: Effect of NAFLD and MC-LR exposure on Body weight and Liver weight; Figure S2: Effect of MC-LR exposure on total body fat in healthy mice; Figure S3: MC-LR exposure significantly increases lipid accumulation in livers of diet-induced NAFLD mice; Figure S4: Reduction in protein carbonyl fragments, a marker of oxidative stress, after antioxidant treatment; Figure S5: Effect of MC-LR exposure on Glutathione-S-transferase (GST) activity; Table S1: Genetic analysis of drug transporters as well as Phase I and II metabolic enzymes in livers from CDHFD-induced NAFLD mice; Table S2: Quantitative PCR analysis of the genetic expression of transporters and enzymes involved in Phase I and II drug metabolism.
