# Supplementary material for: Antioxidant Therapy Significantly Attenuates Hepatotoxicity following Low Dose Exposure to Microcystin-LR in a Murine Model of Diet-Induced Non-Alcoholic Fatty Liver Disease
Source: Antioxidants (Basel). 2022 Aug 22;11(8):1625. doi: 10.3390/antiox11081625 (PMC9404967; doi:10.3390/antiox11081625)
Supplement: Supplementary file 1 [file antioxidants-11-01625-s001.zip › antioxidants-1843526-supplementary.pdf]

Supplementary material:

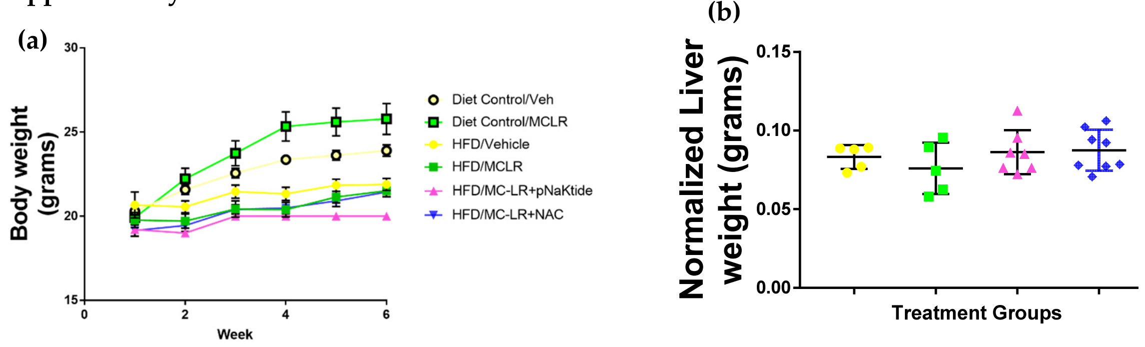

Figure S1: Effect of NAFLD and MC-LR exposure on Body weight and Liver weight. **(a)** Kaplan-Meier analysis of body weights of mice in different groups taken over a period of six weeks revealed that DC mice exposed to the toxin showed an overall increase in body weight as compared to their unexposed counterparts whereas treatment of diet-induced NAFLD mice with pNaKtide after exposure to the toxin showed decrease in body weight as compared to mice that were exposed to the toxin, **(b)** Quantification of liver weights normalized to the body weight did not reveal any significant changes in liver weights of CDHFD-induced NAFLD mice that were exposed to the toxin or those that were treated with antioxidants after exposure to the toxin.

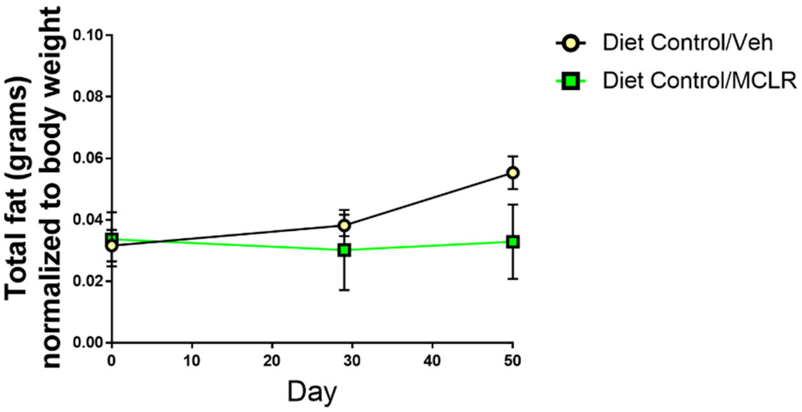

Figure S2: Effect of MC-LR exposure on total body fat in healthy mice. NMR spectroscopy-based analysis revealed no significant alterations in the total body fat content of mice that were exposed to MC-LR.

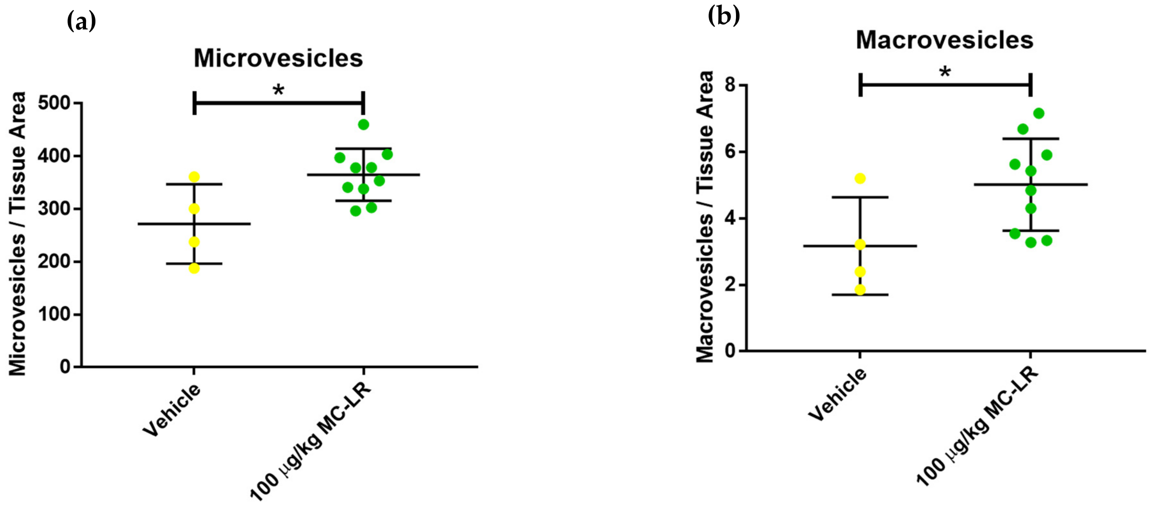

(c)

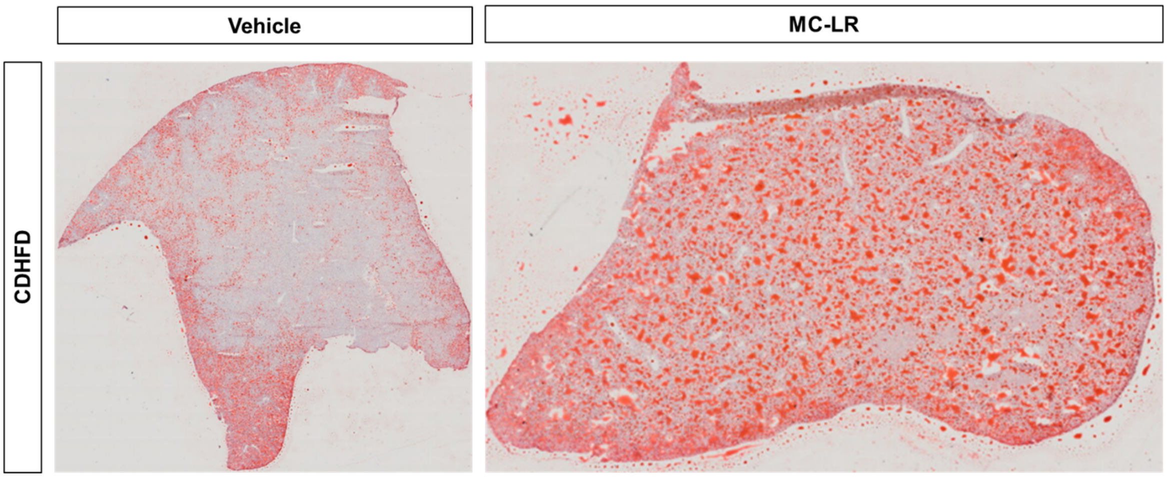

Figure S3: MC-LR exposure significantly increases lipid accumulation in livers of diet-induced NAFLD mice. Histological analysis of livers obtained from diet induced NAFLD mice with or without exposure to the toxin revealed that exposure to even low doses of MC-LR (100 µg/kg) significantly increases lipid accumulation in the form of microvesicles **(a)** and macrovesicles **(b)** within the hepatocytes. **(c)** Representative images of liver tissues stained with Oil Red O stain. The red dye stains the lipid moieties within the tissues. * $p \leq 0.05$.

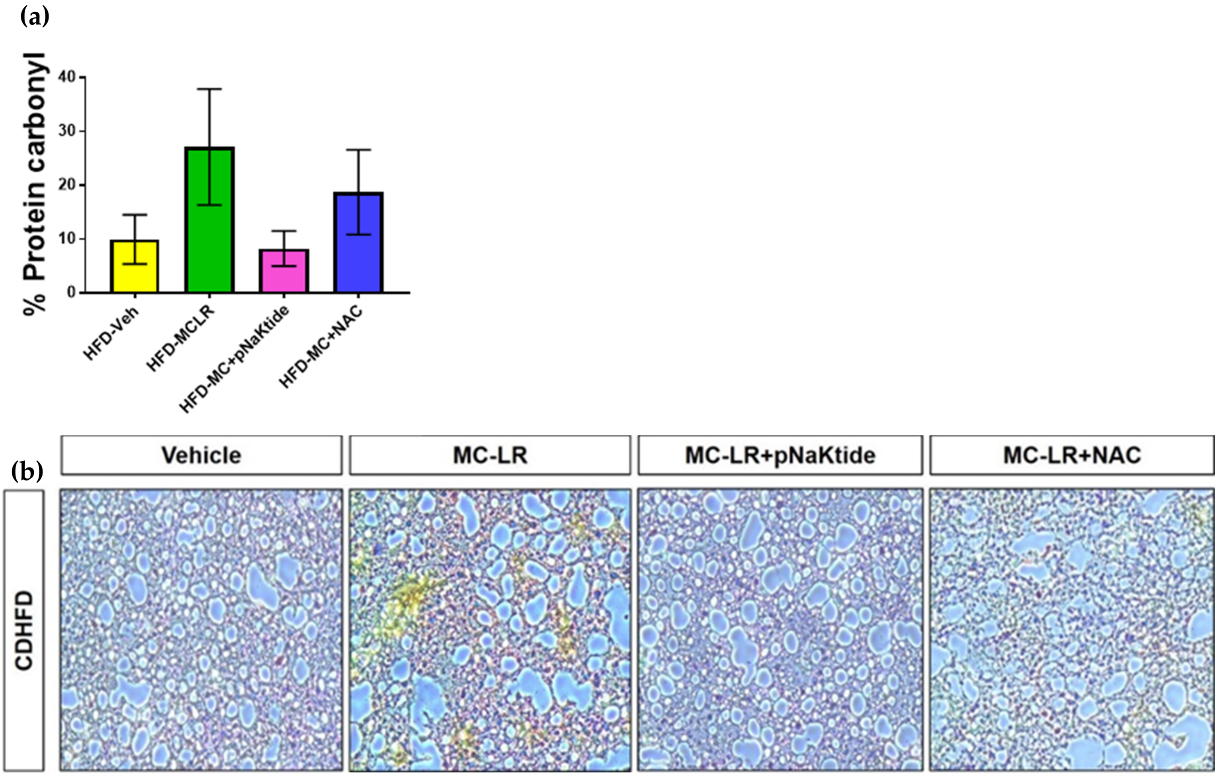

Figure S4: Reduction in protein carbonyl fragments, a marker of oxidative stress, after antioxidant treatment. **(a)** Protein carbonyl levels in liver (n=3) were elevated on exposure to MC-LR and reduced after treatment with antioxidants; **(b)** Representative images of the liver sections stained for assessing the protein carbonyl content.

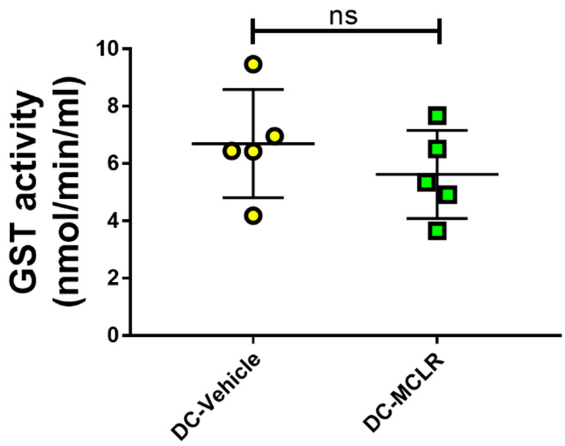

Figure S5: Effect of MC-LR exposure on Glutathione-S-transferase (GST) activity. GST activity was not significantly altered in healthy mice exposed to low doses of MC-LR.

Table S1: Genetic analysis of drug transporters as well as Phase I & II metabolic enzymes in livers from CDHFD-induced NAFLD mice. qPCR arrays were run with liver cDNA from NAFLD mice. Qiagen software was used to run the analysis for different groups. Genes colored in Red were significantly upregulated while those in Blue were significantly downregulated in their respective comparison groups. All of the data presented is significant. ($p \leq 0.05$).

	Effect of MCLR in HFD mice	Effect of antioxidants		Effect of CDHFD	Effect of MCLR in healthy mice	Effect of Antioxidants alone on HFD	
**Drug Transporters**	**HFD-Vehicle vs MCLR**	**HFD-MCLR vs MCLR + pNaKtide**	**HFD-MCLR vs MCLR + NAC**	**DC-Veh vs HFD-Veh**	**DC-Veh vs Treated**	**HFD-Veh vs pNaKtide**	**HFD-Veh vs NAC**
Metallothioneins:	Mt2			Mt3,			
P-Glycoprotein Family Members	Abcb1a	Abcb1a	Gpi1	Abcc1, Gpi			Abcb1a,
**Phase I Metabolizing Enzymes**	Cyp3a11	Cyp3a11	Cyp11b2, Cyp3a11	Cyp17a1, Cyp1a2, Cyp4b1, Cyp2c29, Cyp2e1			Cyp2c29, Cyp2e1, Cyp3a11,
**Phase II Metabolizing Enzymes**							
Carboxylesterases				Ces1g, Ces2c			
Decarboxylases		Gad1	Gad1	Gad1			
Dehydrogenases				Adh1, Adh5		Hsd17b2,	Adh5, Hsd17b2,
Glutathione Peroxidases		Gsta3, Gstm2	Gsta1, Gsta4	Gsta1, Gsta3, Mgst1, Gstm3, Gpx 2, Gpx3, Gstz1, Mpo,	Gpx2, Gstz1, Mpo,		Mpo,

Hydrolases				Ephx1, Ephx2, Faah, Fbp1			
Kinases	Pklr, Pkm	Pklr, Pkm		Hk2, Pklr, Pkm			
Lipoxygenases				Alox12, Apoe			
Oxidoreductases	Blvra	Srd5a1		Aoc1, Cyb5r3, Srd5a1, Blvra, Nos3,	Mthfr	Nos3	Blvra,
Paraoxonases				Pon1			
Glutathione S-Transferases:		Gsta3, Gstm2	Mgst3	Gstm3, Gstt1			Gstt1,
Other Phase II Metabolizing Enzymes				Nat1, Comt, Nat2, Ggt1			
**Other Drug Metabolism Genes**				Gckr, Snn, Arnt			Ahr, Gckr,

Table S2: Quantitative PCR analysis of the genetic expression of transporters and enzymes involved in Phase I & II drug metabolism. qPCR arrays were run with cDNA extracted from the livers of CDHFD diet-induced NAFLD mice exposed to low doses of MC-LR and/or treated with targeted antioxidants such as pNaKtide or NAC. Qiagen software was used to run the analysis for different groups. Numbers in bold indicate significant fold regulation with p value ≤0.05.

		**HFD Veh vs HFD MCLR**		**HFD MCLR vs HFD MCLR+pNakTide**		**HFD MCLR vs HFD MCLR+NAC**		**DC Veh vs DC MCLR**	
**Gene**	**Symbol**	**Fold Regulation**	**p value**	**Fold Regulation**	**p value**	**Fold Regulation**	**p value**	**Fold Regulation**	**p value**
ATP-binding cassette, sub-family B (MDR/TAP), member 1A	Abcb1a	**2.48**	**0.014206**	**-1.82**	**0.031181**	-1.00	0.831654	-1.08	0.907460
ATP-binding cassette, sub-family B (MDR/TAP), member 1B	Abcb1b	1.18	0.492898	1.81	0.413107	1.28	0.523265	1.13	0.756136
ATP-binding cassette, sub-family B (MDR/TAP), member 4	Abcb4	1.00	0.886174	1.14	0.537746	-1.22	0.724269	1.12	0.571156
ATP-binding cassette, sub-family C (CFTR/MRP), member 1	Abcc1	1.22	0.363985	-1.53	0.067772	-1.20	0.278031	-1.02	0.799711
Amiloride binding protein 1 (amine oxidase, copper-containing)	Aoc1	1.91	0.242431	-4.71	0.157960	-1.60	0.390835	-1.94	0.780766
Alcohol dehydrogenase 1 (class I)	Adh1	157.22	0.430152	1.21	0.388849	1.19	0.463936	-1.03	0.788510
Alcohol dehydrogenase 4 (class II), pi polypeptide	Adh4	-1.19	0.603176	1.30	0.506204	-1.24	0.678062	1.06	0.768761
Alcohol dehydrogenase 5 (class III), chi polypeptide	Adh5	-1.04	0.992845	1.17	0.653452	-1.12	0.633176	1.01	0.966724
Aryl-hydrocarbon receptor	Ahr	1.21	0.221962	-1.03	0.808825	-1.03	0.956932	-1.06	0.569601
Aminolevulinate, delta-, dehydratase	Alad	1.09	0.532074	1.01	0.988952	-1.06	0.983574	-1.07	0.734400
Aldehyde dehydrogenase family 1, subfamily A1	Aldh1a1	1.06	0.734233	1.12	0.910483	-1.19	0.844142	1.10	0.513299
Arachidonate 12-lipoxygenase	Alox12	1.21	0.488114	-1.33	0.273823	1.11	0.775093	-1.00	0.993343
Arachidonate 15-lipoxygenase	Alox15	2.21	0.052269	-1.82	0.197424	1.36	0.451231	-4.84	0.377940
Arachidonate 5-lipoxygenase	Alox5	-1.03	0.819576	-1.15	0.560789	1.07	0.992981	-1.06	0.788743
Apolipoprotein E	Apoe	-1.06	0.722594	-1.17	0.529972	1.04	0.764989	1.04	0.670949
Aryl hydrocarbon receptor nuclear translocator	Arnt	-1.19	0.273490	-1.23	0.153904	1.04	0.818846	-1.11	0.650074
ArsA arsenite transporter, ATP-	Asna1	-1.05	0.769962	1.31	0.271494	1.33	0.284325	1.04	0.867214

binding, homolog 1 (bacterial)									
Biliverdin reductase A	Blvra	**1.42**	**0.044023**	-1.34	0.071539	-1.07	0.657063	1.05	0.655463
Biliverdin reductase B (flavin reductase (NADPH))	Blvrb	-1.24	0.386546	-1.05	0.697979	1.08	0.636054	1.11	0.156048
Carboxylesterase 1G	Ces1g	1.11	0.617846	-1.48	0.263278	-1.22	0.632263	-1.25	0.170421
Carboxylesterase 2C	Ces2c	-1.15	0.465117	-1.12	0.560884	1.02	0.871061	-1.05	0.765885
Carbohydrate (keratan sulfate Gal-6) sulfotransferase 1	Chst1	-1.19	0.774899	-1.58	0.541198	1.22	0.611973	-3.31	0.385967
Catechol-O-methyltransferase	Comt	-1.20	0.526019	1.01	0.994706	-1.20	0.491019	-1.16	0.506225
Cytochrome b5 reductase 3	Cyb5r3	-1.36	0.147156	1.08	0.519303	1.10	0.498396	1.17	0.292910
Cytochrome P450, family 11, subfamily b, polypeptide 2	Cyp11b2	-2.35	0.373919	7.59	0.356331	**-42.44**	**0.037352**	1.05	0.659356
Cytochrome P450, family 17, subfamily a, polypeptide 1	Cyp17a1	1.61	0.304172	1.32	0.435738	1.32	0.445055	1.09	0.855740
Cytochrome P450, family 19, subfamily a, polypeptide 1	Cyp19a1	-117.78	0.373901	204.70	0.373869	-1.64	0.507106	-2.08	0.404924
Cytochrome P450, family 1, subfamily a, polypeptide 1	Cyp1a1	1.55	0.201927	1.02	0.908263	-1.03	0.904137	-1.20	0.525868
Cytochrome P450, family 1, subfamily a, polypeptide 2	Cyp1a2	-1.43	0.471695	-1.05	0.835581	-1.37	0.562969	-1.15	0.620745
Cytochrome P450, family 27, subfamily b, polypeptide 1	Cyp27b1	-1.27	0.845770	2.44	0.393394	1.74	0.736693	7.81	0.372150
Cytochrome P450, family 2, subfamily c, polypeptide 29	Cyp2c29	-1.10	0.992385	1.08	0.900408	-1.20	0.531840	1.03	0.881156
Cytochrome P450, family 2, subfamily e, polypeptide 1	Cyp2e1	-1.16	0.716623	1.17	0.669033	1.03	0.965291	1.08	0.540927
Cytochrome P450, family 3, subfamily a, polypeptide 11	Cyp3a11	**1.25**	**0.047157**	**-1.78**	**0.006452**	**-1.82**	**0.004951**	1.27	0.077798
Cytochrome P450, family 3, subfamily a, polypeptide 44	Cyp3a44	2.35	0.429428	-1.70	0.643315	-1.85	0.412536	1.84	0.827338
Cytochrome P450, family 4, subfamily b, polypeptide 1	Cyp4b1	1.04	0.741416	-1.13	0.579111	-1.18	0.657484	-1.35	0.226553
Epoxide hydrolase 1, microsomal	Ephx1	1.11	0.444758	-1.06	0.600954	-1.15	0.570089	-1.14	0.345576
Epoxide hydrolase 2, cytoplasmic	Ephx2	-1.04	0.819633	1.10	0.713964	-1.29	0.647432	1.04	0.799815
Fatty acid amide hydrolase	Faah	1.03	0.918898	1.24	0.291458	-1.12	0.989865	-1.02	0.860994
Fructose bisphosphatase 1	Fbp1	-1.10	0.618928	1.10	0.657683	1.04	0.832326	1.21	0.154303
Glutamic acid decarboxylase 1	Gad1	1.17	0.593998	**-1.91**	**0.044822**	**-2.55**	**0.020720**	5.31	0.406839
Glutamic acid decarboxylase 2	Gad2	-634.73	0.373900	2.61	0.389470	2.79	0.385557	-2.55	0.407364
Glucokinase regulatory protein	Gckr	-1.30	0.202515	1.39	0.135322	1.11	0.688979	-1.07	0.448434

Gamma-glutamyltransferase 1	Ggt1	2.07	0.232062	-1.44	0.456518	-1.02	0.673523	2.13	0.314229
Glucose phosphate isomerase 1	Gpi1	1.17	0.154021	-1.00	0.989949	**1.36**	**0.005406**	-1.07	0.656969
Glutathione peroxidase 1	Gpx1	-1.11	0.531345	-1.26	0.177474	-1.40	0.462801	1.26	0.370408
Glutathione peroxidase 2	Gpx2	1.27	0.468809	-1.19	0.780867	2.95	0.241352	**-9.43**	**0.030818**
Glutathione peroxidase 3	Gpx3	1.52	0.230003	1.16	0.870719	1.34	0.583618	-1.12	0.623949
Glutathione peroxidase 5	Gpx5	-1.54	0.464531	1.00	0.810715	1.39	0.649447	-1.30	0.609346
Glutathione reductase	Gsr	-1.22	0.254823	1.19	0.485369	-1.02	0.945296	-1.15	0.248737
Glutathione S-transferase, alpha 1 (Ya)	Gsta1	-1.06	0.731778	1.19	0.446018	1.67	0.048433	-1.22	0.447502
Glutathione S-transferase, alpha 3	Gsta3	-1.21	0.347458	**1.67**	**0.039196**	1.21	0.483358	1.32	0.110856
Glutathione S-transferase, alpha 4	Gsta4	1.07	0.589160	1.12	0.106586	**1.37**	**0.025898**	1.10	0.613970
Glutathione S-transferase, mu 1	Gstm1	-1.19	0.228344	-1.13	0.183472	-1.05	0.810064	-1.12	0.552566
Glutathione S-transferase, mu 2	Gstm2	-1.25	0.329110	**-1.50**	**0.027929**	-1.34	0.103833	1.01	0.933402
Glutathione S-transferase, mu 3	Gstm3	-1.60	0.125825	1.58	0.128266	1.45	0.348207	-1.53	0.082899
Glutathione S-transferase, mu 4	Gstm4	-1.26	0.234262	1.15	0.518866	-1.04	0.856474	-1.50	0.073864
Glutathione S-transferase, mu 5	Gstm5	1.09	0.755035	-1.12	0.477036	-1.13	0.813840	-1.09	0.519257
Glutathione S-transferase, pi 1	Gstp1	1.40	0.749429	1.75	0.528941	-1.83	0.788107	1.54	0.612684
Glutathione S-transferase, theta 1	Gstt1	1.11	0.508489	1.07	0.803736	1.02	0.885045	1.12	0.328742
Glutathione transferase zeta 1 (maleylacetoacetate isomerase)	Gstz1	-1.10	0.587817	1.10	0.625011	-1.02	0.887067	**1.32**	**0.043819**
Hexokinase 2	Hk2	1.65	0.127754	-1.56	0.071272	1.04	0.648627	1.27	0.180091
Hydroxysteroid (17-beta) dehydrogenase 1	Hsd17b1	-1.16	0.754285	1.22	0.622496	1.89	0.147718	1.45	0.272238
Hydroxysteroid (17-beta) dehydrogenase 2	Hsd17b2	-1.45	0.129534	-1.04	0.970062	-1.38	0.213061	1.05	0.917953
Hydroxysteroid (17-beta) dehydrogenase 3	Hsd17b3	-6.60	0.440560	7.02	0.376877	1.09	0.964794	-1.26	0.760312
Lactoperoxidase	Lpo	-19.65	0.373907	19.48	0.373918	6.53	0.295507	1.21	0.066710
Microsomal glutathione S-transferase 1	Mgst1	-1.30	0.103919	1.12	0.608464	-1.20	0.687807	1.07	0.628826
Microsomal glutathione S-transferase 2	Mgst2	2.07	0.129061	-1.04	0.755358	1.49	0.408084	-1.71	0.465444
Microsomal glutathione S-transferase 3	Mgst3	1.01	0.867439	1.56	0.056947	1.82	0.020271	-1.19	0.527905
Myeloperoxidase	Mpo	-1.52	0.334174	-3.26	0.133234	-1.15	0.646583	**12.34**	**0.002661**
Metallothionein 2	Mt2	**-1.84**	**0.023053**	-1.76	0.067216	-1.17	0.465620	2.17	0.222093
Metallothionein 3	Mt3	-1.26	0.313778	-1.13	0.311319	1.64	0.075628	-1.34	0.206698

5,10-methylenetetrahydrofolate reductase	Mthfr	1.09	0.909503	-1.39	0.212194	-1.57	0.121243	**1.42**	**0.023447**
N-acetyl transferase 1	Nat1	-1.20	0.734220	1.58	0.353253	-1.31	0.410224	1.32	0.255014
N-acetyltransferase 2 (arylamine N-acetyltransferase)	Nat2	1.38	0.281654	1.20	0.499979	1.04	0.755314	1.04	0.758156
Nitric oxide synthase 3, endothelial cell	Nos3	1.34	0.165640	-1.31	0.313638	1.15	0.448792	1.18	0.418327
NAD(P)H dehydrogenase, quinone 1	Nqo1	1.08	0.768371	-1.08	0.711729	-1.19	0.798861	1.36	0.467115
Pyruvate kinase liver and red blood cell	Pklr	**-1.42**	**0.019992**	**1.60**	**0.002698**	1.18	0.529915	-1.28	0.465194
Pyruvate kinase, muscle	Pkm	**1.63**	**0.020729**	**-1.67**	**0.024016**	1.04	0.750995	1.03	0.783285
Paraoxonase 1	Pon1	-1.14	0.847598	1.34	0.489565	1.00	0.991646	-1.01	0.927892
Paraoxonase 2	Pon2	-1.20	0.367056	1.08	0.478359	1.08	0.570160	1.14	0.466434
Paraoxonase 3	Pon3	-1.09	0.625268	1.20	0.460669	1.13	0.610812	-1.11	0.521395
Stannin	Snn	1.25	0.408685	-1.36	0.515546	-1.07	0.690253	-1.09	0.379438
Steroid 5 alpha-reductase 1	Srd5a1	1.10	0.673761	-1.70	0.016144	-1.95	0.068375	-1.04	0.744642
Steroid 5 alpha-reductase 2	Srd5a2	1.22	0.355870	1.67	0.390400	-1.15	0.728459	-1.07	0.721747
Actin, beta	Actb	1.03	0.689732	-1.12	0.422033	-1.15	0.316985	1.07	0.549894
Beta-2 microglobulin	B2m	-1.15	0.285273	-1.20	0.172252	-1.05	0.638436	1.07	0.424956
Glyceraldehyde-3-phosphate dehydrogenase	Gapdh	1.15	0.162047	**1.62**	**0.009275**	1.40	0.067970	1.10	0.399540
Glucuronidase, beta	Gusb	-1.02	0.918994	-1.30	0.148018	-1.23	0.163243	-1.07	0.596780
Heat shock protein 90 alpha (cytosolic), class B member 1	Hsp90ab1	-1.01	0.822693	1.07	0.276644	1.07	0.151802	-1.17	0.271760
